# The enemy within us: lessons from the 2011 European *Escherichia coli* O104:H4 outbreak

**DOI:** 10.1002/emmm.201201662

**Published:** 2012-08-24

**Authors:** Helge Karch, Erick Denamur, Ulrich Dobrindt, B Brett Finlay, Regine Hengge, Ludgers Johannes, Eliora Z Ron, Tone Tønjum, Philippe J Sansonetti, Miguel Vicente

**Affiliations:** 1 Institute of Hygiene, University MünsterMünster, Germany; 2 INSERM, Université Paris DiderotUMR-S 722, Paris, France; 3 Michael Smith Laboratories, Department of Microbiology and Immunology, The University of British ColumbiaVancouver, British Columbia, Canada; 4 Institut für Biologie – Mikrobiologie, Freie Universität BerlinBerlin, Germany; 5 Traffic, Signaling, and Delivery Laboratory, UMR144 Curie/CNRS, Curie InstituteParis, France; 6 Faculty of Life Sciences, Department of Molecular Microbiology and Biotechnology, MIGAL Galil Research Center, Tel Aviv UniversityIsrael; 7 Center for Molecular Biology and Neuroscience, Institute of Microbiology, University of OsloOslo, Norway; 8 Unité de Pathogénie Microbienne Moléculaire et Unité INSERM 786, Institut PasteurParis, France; 9 Centro Nacional de Biotecnología (CNB-CSIC)Madrid, Spain

**Keywords:** *Escherichia coli*, HUS, infection, O104:H4, outbreak

## Abstract

In response to the 2011 European health alert caused by a pathogenic *Escherichia coli* O104:H4 outbreak, the European Academy of Microbiology (EAM), established by the Federation of European Microbiological Societies (FEMS), convened a meeting in Paris on November 30th, 2011 on ‘EHEC infection and control’ attended by world renowned experts in pathogenic *E. coli*. The major aims of this group were to review the scientific issues raised by the outbreak, to assess the handling of the crisis at the scientific and political levels, and to propose future actions. Several conclusions, which will have impact on future potential *E. coli* outbreaks, are outlined here.

## Introduction

**Figure 1:**
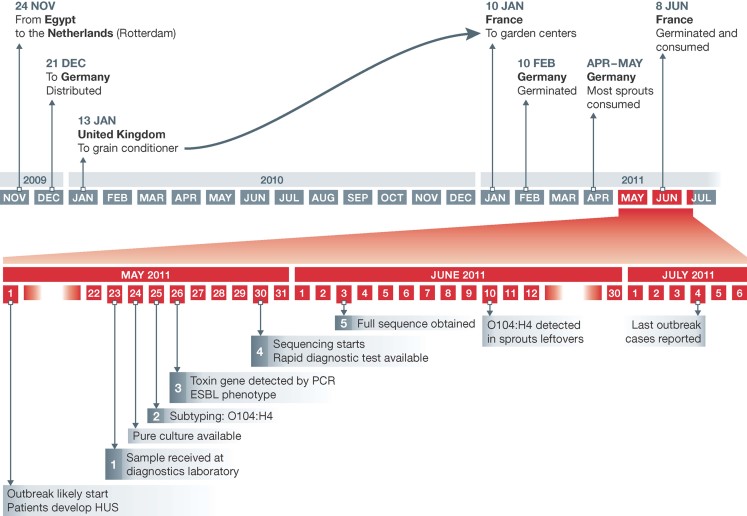
Timecourse of the outbreak The top line shows the retrospective tracing of the fenugreek seed stocks that were linked by association to the German and French outbreak cases. The bottom line (note different scale) indicates significant events occurring at the clinical and diagnostic scenery during the outbreak. No direct connection between the two lines has been firmly established and the circumstances under which the *E. coli* O104:H4 strain came into contact with sprouts have not been documented. (1) Sample received at German National Consulting Laboratory for Hemolytic Uremic Syndrome; (2) Molecular subtyping data available based on partial *gnd*-sequencing (O104), *fliC*-RFLP-typing (H4), MLST (ST678); (3) Stx2 subtype confirmed by PCR and sequencing. ESBL phenotype confirmed; (4) Shotgun genome sequencing on Ion Torrent platform started at German National Consulting Laboratory for HUS. Publication in the Internet of a specific PCR for the differentiation of the outbreak clone from other EHEC; (5) Shotgun genome sequence available.

A major pathogenic *Escherichia coli* O104:H4 outbreak occurred in central Europe during late spring of 2011, infecting nearly 4000 persons mainly in Germany, it produced more than 900 cases of haemolytic uremic syndrome (HUS) resulting in 54 deaths. In addition, the outbreak caused considerable financial losses incurred mainly in unsold produce. A few weeks later a smaller outbreak occurred in southwest France causing 15 cases of bloody diarrhoea of which 9 progressed to HUS. Although identification and characterization of the O104:H4 outbreak strain, including its complete genome sequence, was performed in record time, scientists, as is frequently the case, still do not know exactly how and where it originated (Fig 1). Troubling as this is, the most fearsome aspect of the outbreak is that it may occur again. This deadly bacterium cannot be regarded as a zoonotic disease, its exact natural reservoir is not known and it remains a challenge to farming, agriculture, food safety, public health and scientific research alike. While drugs to combat the disease are largely in very early stages, there is strong consensus among physicians, disease control authorities, and scientists, that shortening the interval to diagnosis and identification of the source of infection is critical. Reliable and responsible communication channels involving clinicians, microbiologists, authorities, media and citizens are also essential to deal with any infectious outbreak.

This position paper reviews the deadly 2011 outbreak from the point of view of molecular microbiology and draws conclusions that might help to prepare for future outbreaks with emphasis on the need to strengthen research efforts in different fields. Research on bacterial evolution, physiology and pathophysiology is needed to complete the basic microbiological description of the disease, while the design of novel diagnostic tools will contribute to characterize the epidemiological aspects. Finally, therapeutic developments, based upon improved knowledge of the mode of action of Shiga toxin (Stx), particularly on endothelial cells in strategically exposed organs such as the kidney and the brain, will help to improve the prospects of a successful treatment of the disease.

## Identification of the O104:H4 strain causing the outbreak

**Figure 2:**
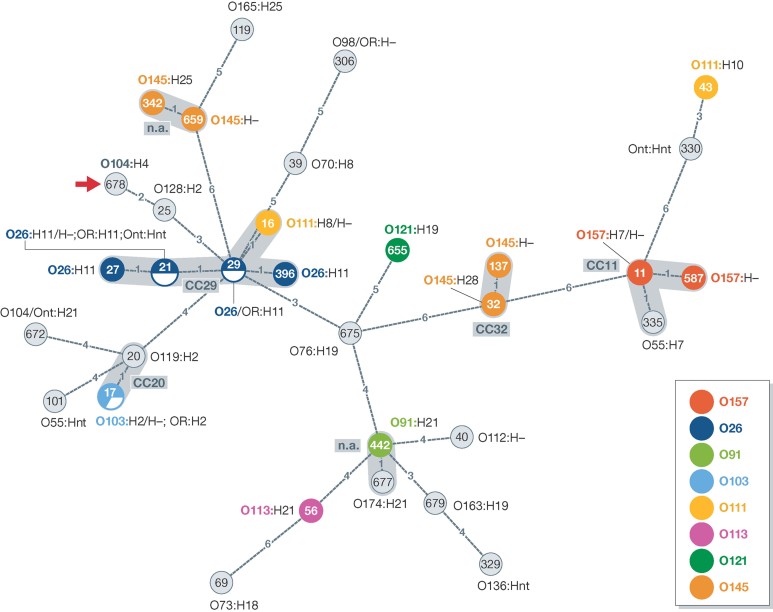
Minimum spanning tree of hemolytic uremic syndrome-associated enterohemorrhagic *E. coli* (HUSEC) strains based on multi-locus sequence typing The red arrow points at the O104:H4 lineage.

Among the handful of *E. coli* strains that inhabit the human intestine, a subset can produce serious disorders frequently associated with the presence of phenotypic traits related to adhesion and toxin production and are often accompanied by antibiotic resistances. The particular strain causing this outbreak showed a combination of virulence factors from both enteroaggregative *E. coli* (EAEC) and enterohaemorrhagic *E. coli* (EHEC) strains (for a review of *E. coli* and its virulence factors see Croxen & Finlay, 2010). Based on its serotype for the O and H antigens, it was classified as O104:H4 (Fig 2).

Infections with O104:H4 had been reported on eight previous occasions, two in Germany (both in 2001), two in France (2004 and 2009) and one each in Korea (2005), Italy (2009), Georgia (2009) and Finland (2010). Usually, both the intimate contact between *E. coli* EHEC strains and the surface of the intestinal cell and the production of effacing lesions are mediated by intimin, encoded by the *eae* gene (Donnenberg et al, 1993). It was therefore surprising to find that the 2011 outbreak isolates were negative for *eae*. Nearly two thirds of the 588 HUS cases, analysed between 1996 and 2010 in Germany by the National Consulting Laboratory HUS, were caused by serogroup O157 (O157:H7, O157:H^−^) and less than 4% were *eae* negative. This means that the 2011 outbreak was caused by a rather infrequent *E. coli* pathogen of unknown origin that was detected in patient stools but not in food.

**Figure 3:**
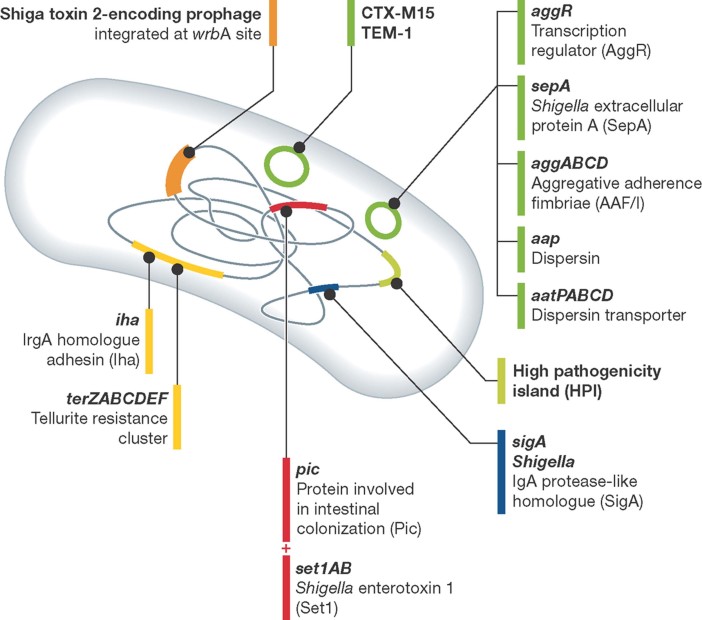
Summary of virulence-associated genes in the genome of the O104:H4 outbreak strain.

One fifth of the German outbreak patients developed HUS. Among the infected patients adults (88%) and young females (68%) were predominant (Frank et al, 2011). In two of the 2001 German infections, HUS had already been associated with EHEC O104:H4 belonging to sequence type (ST) 678 (Mellmann et al, 2008). The 2011 outbreak strain contains the Stx2 encoding gene (Bielaszewska et al, 2011). Several assays demonstrated that this is a hybrid organism that combines some of the virulence genes of EHEC (Stx but not the type III secretion and Tir/intimin system) and EAEC (specially the adherence mechanisms) and expresses the corresponding phenotypes including production of Stx2 and aggregative adherence to cultured intestinal epithelial cells (Bielaszewska et al, 2011; Fig 3). Moreover, this strain shows an extended-spectrum beta-lactamase (ESBL) phenotype. Rapid sequencing of the complete genome from the German clinical isolates was used to fully describe their genetic properties (Brzuszkiewicz et al, 2011; Mellmann et al, 2011; Rasko et al, 2011; Rohde et al, 2011).

## What made this outbreak O104:H4 strain so virulent?

The specific combination of enhanced adhesion, survival fitness, Stx2 production and antibiotic resistance illustrates the high genome plasticity of this *E. coli* pathogen, and helps to explain the high virulence of the outbreak. Expression of Stx2 is co-regulated through induction of an integrated bacteriophage that encodes the toxin gene. At antimicrobial levels above those required to inhibit bacterial replication, several antibiotics such as mitomycin C and quinolones, including ciprofloxacin (Bielaszewska et al, 2012; Rasko et al, 2011), produce DNA damage and therefore induce the SOS response with the unwanted secondary effect of simultaneously triggering phage production and *stx2* gene expression (Kimmitt et al, 2000).

**Figure 4:**
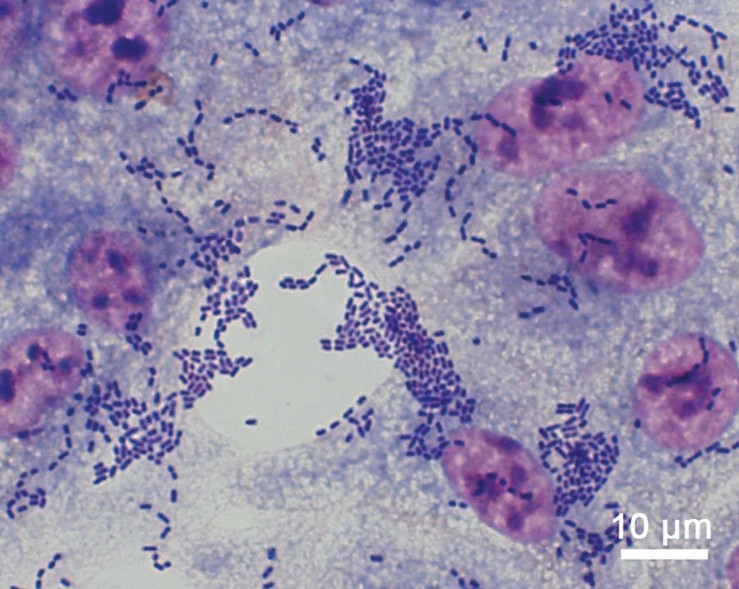
Adherence of the *E. coli* outbreak strain Aggregative ‘stacked-brick’ adherence pattern of the *E. coli* O104:H4 outbreak strain to cultured HCT-8 intestinal epithelial cells.

The increased adherence of the strain to intestinal epithelium, likely mediated by putative adhesins such as aggregative adherence fimbriae I (AAF/I) and other putative adhesins as the iron-regulated gene A homologue adhesin (Iha) and long polar fimbriae (LPF), might facilitate the absorption of Stx2 and could explain the high frequency of patients developing HUS (Bielaszewska et al, 2011; Fig 4). Normally, EHEC Stx delivery is mediated by Tir/intimin adherence, so the unusual combination of an alternate adhesion system (EAEC) with Stx2 could account for the enhanced virulence. Median duration between onset of diarrhoea and development of HUS was 5 days, shorter than the period observed with EHEC O157 (7 days).

The clinical features of the O104:H4 infection were also unusual, including a frequent incidence (nearly 50%) of severe neurological symptoms such as epileptic seizures, paresis, delirium and coma in a subset of HUS patients (Jansen & Kielstein, 2011).

## Origin of the contamination

Tracing back the origin of the outbreak was a difficult and still unresolved challenge, probably because of the very low infection dose. The pathogen was isolated from clinical samples but not from the epidemiologically suspected food vehicle. Epidemiological investigations pointed to salad sprouts as the possible contaminated source (Buchholz et al, 2011; Fig 1). However, no sprouts carrying the specific EHEC strain could be identified in the market. The source of the contamination remained therefore disputed as the EHEC strain could only be identified in leftovers and refuse. Fenugreek sprouts were also a suspected source due to the coincidence between French and German cases. Their origin could be traced back to a common import of seeds from Egypt that arrived to Rotterdam, had been distributed to Germany, and then partly redistributed to the United Kingdom from where a portion finally made its way to France. Nevertheless, the outbreak strain was not present in any of the samples analysed and no contamination was found on farms (European Food Safety Authority, 2011).

## The ancestry of the outbreak strain

Comparison of the genotypes, phenotypes and phylogeny of 80 outbreak isolates demonstrate that the *E. coli* O104:H4 outbreak strain is a clone that combines virulence mechanisms from two different *E. coli* pathotypes: Stx2 producing and enteroaggregative *E. coli* (Fig 2). It is not known when the outbreak strain acquired a plasmid encoding CTX-M-15, an extended-spectrum class A beta-lactamase conferring resistance against ceftazidime (Poirel et al, 2002), which is absent in the original HUSEC041 isolate from 2001 (Mellmann et al, 2011). This plasmid has been identified in several different genera of the *Enterobacteriaceae* family. It is not known when the Stx-producing and enteroaggregative traits in *E. coli* O104:H4 merged in the same progenitor organism.

The German isolates show little diversity, with only two single nucleotide polymorphisms (SNPs) present in isolates from four individuals. A greater diversity (19 SNPs) was found in isolates from seven individuals infected in the French outbreak (Grad et al, 2012). The more homogeneous German isolates form a clade within the more diverse French strains. In the absence of additional data that could explain this diversity between the two sets, it is difficult to determine if diversity was already present in the original set and reduced for unknown reasons in the distribution to Germany, or conversely, if an original homogeneous population was subjected to different mutation rates upon their geographical segregation.

## A human reservoir appears established

In contrast to other EHEC, currently available data suggest that cattle are not the reservoir for the *E. coli* O104:H4 outbreak strain. While it cannot be fully excluded that this strain might have another reservoir, it seems to be adapted to humans (Auvray et al, 2012; Wieler et al, 2011). Indeed, previous data indicated that EAEC are highly adapted to humans, which would suggest that the human population is the reservoir (Harrington et al, 2006; Okeke et al, 2010). Besides casting further doubts on its zoonotic origin, these facts raise the question of potential EHEC O104:H4 carriers, which could shed the pathogen without developing symptoms and therefore, constitute an unsuspected source to further spread the disease among humans.

## The available therapies to cure the disease are not fully effective

Besides intestinal symptoms, the most frequent serious consequence of O104:H4 infection during the outbreak was the progression to HUS linked to the renal lesions caused by Stx. The toxin action results in cell death in the vascular endothelium causing a breakdown of the lining of the blood vessels followed by haemorrhage that is manifested as bloody diarrhoea. Stx is particularly aggressive against small blood vessels, such as those found in the digestive tract, kidneys and lungs. The vascular endothelium of the glomeruli in the kidney is a specific target for the toxin. Destruction of these filtering structures compromises the renal function to the point of causing kidney failure and development of the frequently fatal HUS. http://www.cbwinfo.com/Biological/Toxins/Verotox.html (CBWInfo collection of factsheets on biological and chemical warfare agents). To counteract the disease, the outbreak patients required supportive care to maintain fluid and electrolytes levels, monitor and support kidney function and dialysis to remove the toxin from the bloodstream. Most of them received plasmapheresis, a subset received *eculizumab*, and a few patients underwent immunadsorption (Greinacher et al, 2011).

**Figure 5:**
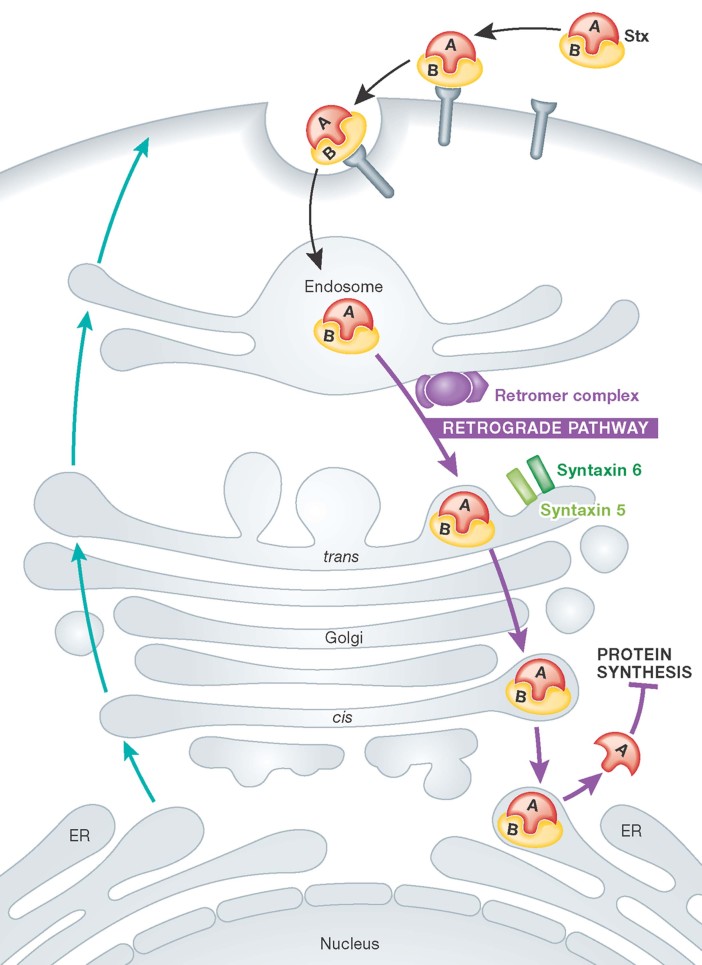
Mode of Shiga toxin action The two component (AB) Shiga Toxin enters the mammalian cell through endocytosis, either mediated or not by clathrin, and is then transported from the endosome to the Golgi network using a section of the secretory pathway. This section, in which a multi-protein retromer complex is needed, is called the retrograde pathway (purple arrows). The toxin then reaches the endoplasmic reticulum (ER) where it is cleaved. The subunit A enters the cytoplasm and blocks protein synthesis at the ribosome, what is the ultimate reason for the toxicity. Small molecule drugs able to selectively block the passage of the toxin through the retrograde pathway to the Golgi network have been found. The toxin is then retained at the endosome where it does not exert virulent effects. Modified from Seaman & Peden (2010).

Stx is formed by two subunits. The A subunit is a *N*-glycosidase able to modify ribosomal RNA. The B subunit, a pentamer, is responsible for attaching the toxin to globotriaosylceramide (Gb3 or CD77) on the cell membrane. Similar to SV40 and other polyoma viruses (Ewers et al, 2010), the binding of Shiga and cholera toxin to their membrane receptors through their common pentameric protein scaffold favours negative membrane curvature (Römer et al, 2007; Fig 5). As a consequence, tubular membrane invaginations lead to the uptake into endosomes where a retrograde endocytic flow directs the toxin into the Golgi and endoplasmic reticulum. The A subunit dissociates from the toxin and is translocated to the cytosol targeting ribosomes where it disrupts protein synthesis resulting in cell death. Although new small-molecule inhibitors that block endosome-to-Golgi retrieval of ricin and Stx protect mice from the deadly effects of the toxins (Mukhopadhyay & Linstedt, 2012; Stechmann et al, 2010), they are not available for clinical use.

Antibiotics are of little help in treating most intestinal infections and in fact can be contraindicated in EHEC infections (Tarr et al, 2005; Wong et al, 2012) as they can promote the induction of temperate bacteriophages carrying the Stx encoding genes, in the case of inhibitors of DNA replication, or induce the release of higher amounts of the toxin itself for those as beta-lactams that result in cell lysis (Kimmitt et al, 2000). In principle, most antibiotics, independent of their mode of action, can trigger the SOS response (global response to DNA damage; Storz & Hengge, 2011) to some extent, and therefore their use may eventually worsen the state of the patients. Via these effects antibiotics may increase the risk of progression of an EHEC infection into HUS (Tarr et al, 2005). A recent study, which investigated effects of various antibiotics on induction of Stx2-encoding bacteriophages and Stx2 production in the *E. coli* O104:H4 outbreaks strain demonstrated that, at sub-inhibitory concentrations, ciprofloxacin significantly increased induction of the toxin-encoding phage and Stx2 production. On the contrary, other antibiotics such as meropenem, azithromycin, rifaximin and tigecycline did not influence the phage and toxin levels or even decreased their production *in vitro* (Bielaszewska et al, 2012). However, further studies in animal models, as well as careful analyses of clinical outcomes in patients who were treated with these antibiotics during the German outbreak, are necessary to ultimately assess their potential usefulness for treatment of humans infected with EHEC O104:H4, if antibiotic therapy is necessary.

The severity of the outbreak brought to light the existence of a humanized monoclonal antibody against complement protein C5 that inhibits activation of the terminal complement pathway. Activation of this pathway, leading to cell damage and tissue destruction, also occurs in the uremic syndrome caused by the STEC strains. The antibody, produced under the name eculizumab, was developed as an experimental drug to treat paroxysmal nocturnal haemoglobinuria, a seemingly unrelated disease. Eculizumab had been used successfully to treat atypical (Chatelet et al, 2009) and EHEC associated HUS (Lapeyraque et al, 2011).

## The impact on the population and the social cost

Compared to other infectious diseases like tuberculosis, malaria or HIV, the number of persons affected by the 2011 European *E. coli* O104:H4 outbreak can certainly be defined as minor. This contrasts with the alarm perceived by the public opinion and the extensive coverage received in the media, particularly in Europe. The somehow disproportionate public response could be attributed to the facts that the pathogen was foodborne and caused a surprisingly high number of fatalities while health authorities could not identify the source of contamination. Together, these facts created significant uneasiness and exposed the perhaps false assumption that modern affluent societies nowadays are almost free from the risk of major bacterial infections. During the weeks in which the cases were most frequent, the number of worldwide accessions through Google for information on EHEC was extremely high approaching the celebrity status. This number peaked when cases were first reported in other European countries outside Germany (http://guusroeselers.blogspot.com/2011/05/ehec.html).

By attempting to restrain damage, but acting on limited knowledge, some local authorities warned about possible sources of contaminated produce that in the end were not genuine. Initial reports linking the German outbreak to Spanish vegetables were not ultimately confirmed. Despite the serious setback in the production of Spanish vegetables during the outbreak alert, they tested negative for the O104:H4 serotype (Mora et al, 2011). The exact economic impact on Spanish agricultural production is difficult to calculate. Spanish authorities estimated the direct damages caused by produce withdrawal in at least 51 million Euros while nearly 200 million Euros in production loss have been claimed (http://www.elpais.com/articulo/economia/Aguilar/cifra/51/millones/danos/crisis/pepino/elpepueco/20110627elpepueco_9/Tes). In addition, all the health care expenditure on patient diagnostics and epidemiological analyses, hospitalization, dialysis and future renal organ transplantations must be included as long-term societal expenses.

A simple calculation indicates that nearly 10^20^ *E. coli* cells are shed *per* day into the environment by a world population of more than six billions (10^10^ per person). While this number illustrates the threat posed by potential outbreaks of pathogenic *E. coli*, the main danger of new potential outbreaks is that we are not in a position to predict how lethal they may become. At least 133 hot spots in which insertions of genetic material can be sustained have been identified in the *E. coli* chromosome. This confers a high level of plasticity to this bacterial genome that can result in genomic rearrangements yielding novel combinations with a high potential to develop virulent variations (Denamur, 2011; Touchon et al, 2009).

## What could be improved to address future outbreaks?

Progress in the discovery and development of drugs to block the action of the Stx appears as the most potent and promising treatment to alleviate the disease. New antibiotics that do not trigger a SOS response and do not lead to extensive lysis of the pathogen could also offer tools to curb the infection, as would molecules that compete with bacterial attachment to the intestinal mucosa. The development of these alternatives by sustaining long-term basic and applied research programs focused on microbiology and related disciplines is heavily dependent on the allocation of resources; success may be therefore threatened by budgetary cuts.

Vaccination is a preventive tool that is being used to counteract *E. coli* O157:H7 infections, specifically to reduce shedding in cattle, which are asymptomatic carriers of this strain and are considered its main reservoir. Reducing cattle shedding may offer a significant protection for humans, as preventing the direct contamination of meat and dairy products and the indirect passage of the pathogen to vegetables through contaminated irrigation waters would affect the main sources of human infection. Pilot vaccination programmes with a vaccine containing type III secreted proteins, Tir and EspA, involved in bovine colonization significantly reduced bacterial shedding by the vaccinated cows and a protective vaccine is already commercially available in Canada (Allen et al, 2011). Development of a similar vaccination scheme for O104:H4 does not appear feasible at this time given the absence of solid data identifying the primary reservoir responsible for human exposure to *E. coli* O104:H4, as well as any latent reservoirs of this pathogen in the environment.

Another preventive action involves the study of the exact, and up to now unknown, pathways leading to the acquisition of pathogenic traits by *E. coli*, and the selective pressures that may lead to their consolidation in a new virulent and potentially lethal strain. It may require a great deal of sustained effort, in terms of resources and research manpower, to expand the available knowledge in microbial ecology and to obtain an exhaustive description of the global regulatory pathways operating in bacteria (Storz & Hengge, 2011) in order to design potent predictive tools. It is difficult to predict the variety of permutations and combinations of the many *E. coli* virulence factors and their potential to cause disease. It is also possible that new genes encoding virulence factors could be acquired from other bacterial pathogens.

Meanwhile public health authorities should be prepared to monitor the cases of serious intestinal infections with particular emphasis on those acquired by travellers to exotic destinations, as their exposure to unsuspected pathogenic recombinant strains may be high. Tests such as PCR typing and immunodetection of Stx in stools may help early diagnosis and contribute to improve the outcome of the disease in patients. Although costly, these measures can greatly reduce fear and stress and even translate into long-term savings if they are effective in avoiding major clinical consequences, *e.g.* kidney failure requiring organ transplantation or life-long dialysis after a HUS episode.

Since the 2011 *E. coli* O104:H4 outbreak the adequacy of the available collective base of scientific and medical knowledge regarding potentially lethal *E. coli* pathogens has been questioned. The way in which information about the outbreak and the identification of the pathogen was conveyed to the public was also debated. Handling an effective and reliable flow of information between health professionals, scientists, media, politicians and finally the public is certainly a complex and delicate issue. Better communication between clinicians and microbiologists would be a first step towards preventing waste of precious time. Once an outbreak of pathogenic bacteria is detected, the time elapsed from the onset of clinical symptoms and the full molecular typing of the pathogen can be critical to improve the prognosis of the disease and to curb its spread to the population. Although the German outbreak samples were immediately sent to local diagnostic laboratories and an EHEC was detected, the exact strain typing (*e.g.* O antigen and flagellar serotype, sequence type) could not be determined.

The initial political handling of the 2011 outbreak was based on incomplete data that later proved to be incorrect, but nevertheless caused a negative economic impact on the European agricultural industry. Economic losses were registered in Spain, as the initial warning of the authorities focused on Spanish produce, but also affected other countries, including Belgium, Bulgaria, France, Portugal, Switzerland, The Netherlands and Germany. The crisis modified the habits of the consumers regarding the inclusion of fresh vegetables in their diet following the German government recommendation to avoid eating raw cucumbers, tomatoes, lettuce and sprouts. The handling of the information by the politicians is a rather a sensitive point because in addition to considerations on political opportunity and partisan gain, the authorities need to strike a balance between the need to alert the population to avoid undesired and preventable casualties and the cautious behaviour required to prevent panic. Moreover, these decisions often have to be adopted as new data become available. A similar reasoning can be applied to how the media needs to handle this information. At the root of this question is the way in which Science deals with problems, providing plausible hypotheses and assigning probabilities in a continuous process of knowledge acquisition. While this is not the sort of clear-cut facts that politicians, journalists and the public usually demand, scientists could help by establishing channels to provide timely and scientifically reliable information that can be conveyed in a language accesible to the layman. In retrospect, this approach was not fully satisfactory with the handling of the 2011 *E. coli* O104:H4 outbreak.

One of the many lessons of this outbreak is that *E. coli* has an exquisite mastery to acquire and combine genes that may convert the pathogen into an insidious one, able to subvert the physiology of human cells. It is not at all clear that humans are equally clever at counteracting or even at detecting the bacteria and preventing its rapid spread.
